# Effects of Deletion of Macrophage ABCA7 on Lipid Metabolism and the Development of Atherosclerosis in the Presence and Absence of ABCA1

**DOI:** 10.1371/journal.pone.0030984

**Published:** 2012-03-05

**Authors:** Illiana Meurs, Laura Calpe-Berdiel, Kim L. L. Habets, Ying Zhao, Suzanne J. A. Korporaal, A. Mieke Mommaas, Emmanuelle Josselin, Reeni B. Hildebrand, Dan Ye, Ruud Out, Johan Kuiper, Theo J. C. Van Berkel, Giovanna Chimini, Miranda Van Eck

**Affiliations:** 1 Division of Biopharmaceutics, Leiden/Amsterdam Center for Drug Research, Gorlaeus Laboratories, Leiden University, Leiden, The Netherlands; 2 Current position at Department of Endocrinology and Metabolic Diseases, Leiden University Medical Center, Leiden, The Netherlands; 3 Electron Microscopy Section, Department of Molecular Cell Biology, Leiden University Medical Center, Leiden, The Netherlands; 4 Centre d'Immunologie de Marseille Luminy, Institut National de la Santé et de la Recherche Médicale, Université de la Méditerranée, Marseille, France; Cardiovascular Research Institute Maastricht - Maastricht University, The Netherlands

## Abstract

ABCA7, a close relative of ABCA1 which facilitates cholesterol efflux to lipid-poor apoproteins, has been implicated in macrophage lipid efflux and clearance of apoptotic cells in *in vitro* studies. In the current study, we investigated the *in vivo* effects of macrophage ABCA7 deficiency on lipid metabolism and atherosclerosis. Chimeras with dysfunctional ABCA7 in macrophages and other blood cells were generated by transplantation of bone marrow from ABCA7 knockout (KO) mice into irradiated low-density lipoprotein receptor (LDLr) KO mice. Unexpectedly, macrophage ABCA7 deficiency did not significantly affect atherosclerosis susceptibility of LDLr KO mice after 10 weeks Western-type diet feeding. However, ABCA7 deficiency was associated with 2-fold (p<0.05) higher macrophage ABCA1 mRNA expression levels. Combined disruption of ABCA1 and ABCA7 in bone-marrow-derived cells increased atherosclerotic lesion development (1.5-fold (p>0.05) as compared to wild type transplanted mice. However, single deletion of ABCA1 had a similar effect (1.8-fold, p<0.05). Macrophage foam cell accumulation in the peritoneal cavity was reduced in ABCA1/ABCA7 dKO transplanted animals as compared to single ABCA1 KO transplanted mice, which was associated with increased ABCG1 expression. Interestingly, spleens of ABCA1/ABCA7 double KO transplanted mice were significantly larger as compared to the other 3 groups and showed massive macrophage lipid accumulation, a reduction in CD3+ T-cells, and increased expression of key regulators of erythropoiesis. In conclusion, deletion of ABCA7 in bone marrow-derived cells does not affect atherogenesis in the arterial wall neither in the absence or presence of ABCA1. Interestingly, combined deletion of bone marrow ABCA1 and ABCA7 causes severe splenomegaly associated with cellular lipid accumulation, a reduction in splenic CD3+ T cells, and induced markers of erythropoeisis. Our data indicate that ABCA7 may play a role in T cell proliferation and erythropoeisis in spleen.

## Introduction

Reverse cholesterol transport (RCT), defined as the transport of accumulated cholesterol from peripheral tissues back to the liver for biliary excretion, plays an important protective role in atherogenesis [1], [2]. In this process, cholesterol efflux represents a crucial mechanism to maintain cellular lipid homeostasis in macrophages and to prevent pathological foam cell formation, a hallmark of atherosclerosis. A key regulator of macrophage cholesterol and phospholipid efflux is Adenosine-triphosphate-Binding Cassette (ABC) transporter ABCA1. ABCA1 promotes the first step in RCT, namely the efflux of cholesterol and phospholipids to lipid-poor apolipoprotein such as apolipoprotein (apo) A-I, thereby initiating the generation of high-density lipoprotein (HDL) [3], [4], [5]. Mutations in the ABCA1 gene are the underlying cause of Tangier disease in humans, characterized by intracellular cholesterol accummulation and severe HDL-deficiency associated with cardiovascular disease [6], [7], [8], [9]. In addition to ABCA1, macrophage ABCG1 also plays a significant role in macrophage lipid homeostasis by inducing cellular cholesterol efflux to mature HDL [10], [11].

Several other ABC transporters are expressed in monocyte-derived macrophages and have been suggested to be involved in lipid transport processes, RCT, and atherosclerosis [12], [13]. Amongst them is ABCA7, which is a very close relative of ABCA1 (54% homology; both ∼220 kDa) and has been anticipated to play a role in cellular cholesterol homeostasis [14]. ABCA7, a full-size ABC transporter, is detected in both the plasma membrane and in intracellular compartments and is expressed predominantly in myelo-lymphatic tissues with highest expression in peripheral leukocytes, thymus, spleen, and bone marrow [14], [15].

Interestingly, computer-based analysis of the ABCA7 genomic region upstream of the transcriptional start site revealed multiple potential binding sites for transcription factors with roles in hematopoiesis, in particular in the development and activation of cells of the myeloid lineage [16]. While not yet confirmed experimentally, this finding is consistent with the preferential distribution of ABCA7 in hematopoietic tissues.

Expression of ABCA7 is induced during *in vitro* differentiation of human monocytes into macrophages and it is reported to be a sterol-sensitive gene [14], [17]. Both the ABCA7 mRNA and protein expression in human macrophages were upregulated by acetylated low-density lipoprotein (LDL) loading and downregulated under HDL deloading conditions [14]. In macrophages, ABCA7 thus exhibits a similar regulatory response to cholesterol influx and efflux as ABCA1 and ABCG1 [18], [19]. In contrast, Iwamoto *et al*. [17] reported that in mouse embryonic fibroblasts, ABCA7 is upregulated by cellular cholesterol depletion and downregulated upon cholesterol loading through the sterol regulatory element binding protein (SREBP) 2 pathway.

Human ABCA7 mediates the apolipoprotein-dependent generation of HDL by releasing both cellular cholesterol and phospholipids in transfected HEK293 cells [20], [21]. Likely, another study reported that ABCA7 functions as a lipid transporter with efflux specificity for phospholipids over cholesterol in HEK293 cells overexpressing ABCA7 [22]. However, genetic knockdown of ABCA7 by two different approaches in mouse peritoneal macrophages did not affect phospholipid or cholesterol efflux activity to apoA-I [23]. Accordingly, macrophages isolated from ABCA7 null mice displayed a normal apolipoprotein-mediated capacity to efflux both phosphatidylcholine and cholesterol *in vitro* [24]. All together, ABCA7 has been implicated in several physiologically important pathways, nevertheless its role in macrophage foam cell formation in vivo and atherosclerosis remains unknown. Therefore, the goal of the present study was to gain insight into the role of ABCA7 in atherosclerotic lesion development. Since ABCA7 deficiency in macrophages led to a significant up-regulation of ABCA1, we compared the effects of combined macrophage ABCA1 and ABCA7 deletion with single macrophage deficiencies of these transporters on lipid metabolism and atherosclerosis *in vivo* by means of the bone marrow transplantation (BMT) strategy.

## Materials and Methods

### Animals

ABCA7 knockout (ABCA7 KO) mice were generated and kindly provided by Dr. M.W. Freeman (Massachusetts General Hospital, Boston, USA). ABCA1 knockout (ABCA1 KO) and ABCA7 KO mice were backcrossed onto a pure C57BL/6 background for >12 and >7 generations, respectively, and cross-bred to generate ABCA1/A7 double knockout (dKO) mice at the Centre d'Immunologie de Marseille Luminy. Homozygous LDL receptor knockout (LDLr KO) mice were obtained from The Jackson Laboratory (Bar Harbor, Me) as mating pairs and bred at the Gorlaeus Laboratory (Leiden, The Netherlands). Mice were housed in sterilized filter-top cages in a temperature-controlled room with a 12-h light/dark cycle and food and water were provided *ad libitum*. Mice were maintained on sterilized regular chow, containing 4.3% (w/w) fat and no cholesterol (RM3, Special Diet Services, Witham, UK) or fed Western-type diet (WTD) to induce atherosclerosis, containing 15% (w/w) fat and 0.25% (w/w) cholesterol (Diet W, Abdiets, Woerden, The Netherlands). Drinking water was supplied with antibiotics (83 mg/L ciprofloxacin and 67 mg/L polymyxin B sulfate) and 6.5 g/L sucrose. Animal experiments were performed at the Gorlaeus Laboratories of the Leiden/Amsterdam Center for Drug Research in accordance with the National Laws (ID 04081.1). All experimental protocols were approved by the Ethics Committee for Animal Experiments of Leiden University and carried out in compliance with the Dutch government guidelines.

### Bone marrow transplantation (BMT)

To induce bone marrow aplasia, LDLr KO recipient mice of 10–12 weeks old were exposed to a single dose of 9 Gy (0.19 Gy/min, 200 kV, 4 mA) total body irradiation using an Andrex Smart 225 Röntgen source (YXLON International, Hamburg, Germany) with a 6-mm aluminum filter 1 day before the transplantation (n = 14–15 per group). Bone marrow was isolated by flushing the femurs and tibias from the donor ABCA7 KO and C57BL/6 wild type (WT) (first BMT) or ABCA1 KO, ABCA7 KO, dKO and WT (second BMT) mice with phosphate-buffered saline (PBS). Single-cell suspensions were obtained by passing the cells through a 70 µm cell strainer (Falcon, The Netherlands). Irradiated recipients received 5×10^6^ bone marrow cells by intravenous injection into the tail vein. After a BMT recovery period of 8 weeks mice were fed WTD for 10 weeks, after which animals were sacrificed. Body mass was recorded weekly throughout the study. At the end of the study successful reconstitution of recipient mice with donor bone marrow was confirmed by analysis of ABCA1 and ABCA7 transcripts in genomic DNA from bone marrow of the transplanted mice.

### Assessment of successful bone marrow reconstitution

The hematologic chimerism of the LDLr KO mice was determined using genomic DNA from bone marrow by polymerase chain reaction (PCR) at 18 weeks after transplant. The forward and reverse primers 5′-TGGGAACTCCTGCTAAAAT-3′ and 5′-CCATGTGGTGTGTAGACA-3′ for mouse endogenous ABCA1 gene; 5′-TTTCTCATAGGGTTGGTCA-3′ and 5′-TGCAATCCATCTTGTTCAAT-3′ for mouse targeted ABCA1 gene; 5′-CTGCTCAGCTACAGCCTGTGG-3′, 5′-CAT GATGACCACACGAGAGCC-3′ and, 5′-CGGATCCGCTGTAAGTCTGCA-3′ for mouse endogenous and targeted ABCA7 gene were used.

### Serum lipid and lipoprotein analyses

After an overnight fast, ≈100 µL of blood was drawn from each mouse by tail bleeding. The concentrations of total cholesterol in serum were determined using enzymatic colorimetric assays as described previously [25]. The concentrations of phospholipids and triglycerides in serum were determined using enzymatic colorimetric assays (Spinreact S.A., Girona, Spain and Roche Diagnostics, Mannheim, Germany, respectively). Precipath I (Roche Diagnostics, Mannheim, Germany) was used as an internal standard. Absorbance was read at 490 nm. The distribution of lipids over the different lipoproteins in serum was determined by fractionation of 30 µl serum of each mouse using a Superose 6 column (3.2×300 mm, Smart-system, Pharmacia, Uppsala, Sweden). Cholesterol contents in the effluent were determined as above. After 10 weeks WTD feeding, total blood cells were quantified using an automated Sysmex XT-2000iV analyzer (Goffin Meyvis, Etten-Leur, The Netherlands).

### Lipid extraction of peritoneal leukocytes and spleen

Liver lipids were extracted with isopropyl alcohol–hexane as described [26], dried with nitrogen, reconstituted with isopropyl alcohol–0.5% sodium cholate, and sonicated for 10 min (50 Hz) on ice, prior to lipid measurements.

### Analysis of peritoneal leukocytes

After 10 weeks WTD feeding, the peritoneal cavity of 6–7 mice per group were lavaged with 10 ml cold PBS to collect non-thioglycollate stimulated peritoneal leukocytes for quantification of macrophage foam cells using an automated Sysmex XT-2000iV analyzer (Goffin Meyvis, Etten-Leur, The Netherlands). Corresponding samples were cytospun using the Thermo Shandon Cytospin 4 (5 min at 500 rpm) for manual confirmation and stained with Oil red O for detection of lipid accumulation.

### Flow cytometry analysis

After 10 wks of WTD feeding, the mice (not thioglycollate stimulated) were sacrificed and blood, spleen, liver, lymph nodes, thymus, bone marrow and peritoneal macrophages were isolated (5 per group). Single cell suspensions were obtained by mashing the cells trough a 70 µm cell strainer (Falcon, The Netherlands). Red blood cells in blood, bone marrow and spleen samples were lysed using 0.83% NH_4_Cl in 0.01 M Tris/HCl, pH 7.2. Mononuclear cells from the liver were isolated using Lympholyte (Cedarlane Laboratories, Ontario, Canada). Subsequently, 300.000 cells were stained with the appropriate antibodies (eBioscience, Halle-Zoersel, Belgium) to determine cell subsets of interest: T cells (CD3^+^ (total), CD4^+^ (T helper), CD8^+^ (cytotoxic), CD4^+^CD25^high^ (regulatory), CCR7 (homing of cells to lymphoid organs), TCRβNK1.1 (natural killer), monocytes (CD14^+^) and B cells (CD19^+^), macrophages (F4/80^+^, CD11b^+^, CD23^+^F4/80^+^ (M1 class), CD23^−^F4/80^+^CD86^+^ (M2 class)), dendritic cells (CD11c^+^, MHCII^+^, CD86^+^). FACS analysis was performed on the FACSCalibur (Becton Dickinson, Mountain View, CA, USA). Data were analyzed using Cell Quest software.

### Macrophage cholesterol efflux studies

After 10 weeks WTD feeding, 5–7 mice per group were injected with 3% Brewer's thioglycollate medium to induce the infiltration of macrophages into the peritoneal cavity. At 5 days after injection, the peritoneal cavity of the mice was lavaged with 10 mL cold PBS to collect thioglycollate-elicited peritoneal leukocytes. Subsequently, thioglycollate-elicited peritoneal macrophages were incubated with 0.5 µCi/mL ^3^H-cholesterol in DMEM/0.2% BSA (fatty acid free) for 24 hours at 37°C. To determine cholesterol loading, cells were washed 3 times with washing buffer (50 mmol/L Tris containing 0.9% NaCl, 1 mmol/L EDTA, and 5 mmol/L CaCl_2_, pH 7.4), lysed in 0.1 mol/L NaOH, and the radioactivity was determined by liquid scintillation counting. Cholesterol efflux was studied by incubation of the cells with DMEM/0.2% BSA alone or supplemented with either 10 µg/mL apoA-I (Calbiochem, San Diego, USA) or 50 µg/mL human HDL (density 1.063 to 1.21 g/mL, isolated according to Redgrave et al.) as lipid acceptors. Radioactivity in the medium and the cells was determined by scintillation counting after 24 hours of incubation. Cholesterol efflux was calculated as the amount of radioactivity in the medium compared to the total amount of radioactivity measured in medium plus cells.

### Histological and tissue lipid analysis

To analyze lipid accumulation in the different tissues, mice were sacrificed after 10 weeks (18 weeks after BMT) on WTD. After *in situ* perfusion, organs were excised and stored in 3.7% neutral-buffered formalin (Formal-fixx; Shandon Scientific Ltd, UK). Subsequently, cryosections of formalin-fixed lung, liver, mesenteric lymph nodes, spleen, and the aortic root in the heart were prepared from all groups of mice and stained for lipid accumulation using Oil red O. Hematoxylin (Sigma Diagnostics, St. Louis, MO, USA) was used to stain the nuclei in the different organs. Atherosclerotic mean lesion area (in µm^2^) was quantified in the aortic root from ten Oil red O-stained sections for each animal, starting at the appearance of the tricuspid valves. Corresponding sections of atherosclerotic lesions and the spleen were stained immunohistochemically with antibodies against macrophage F4/80 specific antigen (polyclonal rat IgG2b, Research Diagnostics Inc, NJ) or against lymphocyte CD3 antigen (SP7)(NeoMarkers, Fremont, CA). Apoptotic cells in atherosclerotic lesions and spleen cells were identified by terminal deoxynucleotidyl transferase-mediated dUTP nick end-labeling (TUNEL) staining using the In Situ Cell Death Detection Kit (Roche Diagnostics, Mannheim, Germany). TUNEL-positive nuclei were visualized with Nova Red (Vector Laboratories, Burlingame, CA, USA), and sections were counter stained with 0.3% methylgreen. The level of apoptosis was expressed as the percentage of TUNEL-positive cells and as amount of TUNEL-positive cells per µm^2^ lesion area. Sections pre-treated with DNase (2 U per section) enabled labeling of all cells and served as positive control. A polyclonal rabbit anti Ki67 antibody (Abcam, Cambridge, UK) was used to determine spleen cell proliferation rate. A rat TER 119 antibody (Santa Cruz Biotechnology Inc., Germany) was used as the primary antibody to immunohistochemically stain spleen erythroid lineage of mouse origin [27]. All quantifications were performed blinded by using the Leica image analysis system, consisting of a Leica DMRE microscope coupled to a video camera and Leica Qwin Imaging software (Leica Ltd., Cambridge, UK).

### Electron microscopy

Spleens were fixed in 3.7% neutral-buffered formalin (Formal-fixx; Shandon Scientific Ltd, UK), post-fixed in 1% OsO_4_ in the same buffer for 1 hour at 4°C, dehydrated in a graded ethanol series and embedded in Epon. Ultrathin sections were post-stained with uranyl acetate and lead citrate and viewed with a CM10 electron microscope at 80 kV (Philips, Eindhoven, The Netherlands).

### Gene mRNA and protein expression analysis

Guanidium thiocyanate-phenol was used to extract total RNA from peritoneal macrophages and spleens. cDNA was generated using RevertAid M-MuLV reverse transcriptase (Fermentas, Burlington, Canada) according to manufacturer's protocol. Quantitative gene expression analysis was performed using the SYBR-Green method on a 7500 fast Real-time PCR machine (Applied Biosystems, Foster City, CA). PCR primers (**Table 1**) were designed using Primer Express Software according to the manufacturer's default settings. Hypoxanthine Guanine Phosphoribosyl Transferase (HPRT), β-actin, and acidic ribosomal phosphoprotein PO (36B4) were used as the standard housekeeping genes. Relative gene expression was calculated by subtracting the threshold cycle number (Ct) of the target gene from the average Ct of housekeeping genes and raising two to the power of this difference, in order to exclude the possibility that changes in the relative expression were caused by variations in expression of a single housekeeping gene.

**Table 1:** Primers used for quantitative real-time RT-PCR analysis.

Gene	GeneBank Accession	Forward primer	Reverse Primer
**β-actin**	X03672	AACCGTGAAAAGATGACCCAGAT	CACAGCCTGGATGGCTACGTA
**36B4**	X15267	GGACCCGAGAAGACCTCCTT	GCACATCACTCAGAATTTCAATGG
**HPRT**	J00423	TTGCTCGAGATGTCATGAAGGA	AGCAGGTCAGCAAAGAACTTATAG
**ABCA1**	NM_013454	GGTTTGGAGATGGTTATACAATAGTTGT	TTCCCGGAAACGCAAGTC
**ABCG1**	NM_009593	AGGTCTCAGCCTTCTAAAGTTCCTC	TCTCTCGAAGTGAATGAAATTTATCG
**CD3**	NM_007648.3	CTGTCTAGAGGGCACGTCAA	GATGCGGTGGAACACTTTCT
**CD4**	NM_013488	GAGCTCTTGTTGGTTGGGAA	CGAACATCTGTGAAGGCAAA
**CD68**	NM_009853	CCTCCACCCTCGCCTAGTC	TTGGGTATAGGATTCGGATTTGA
**CD19**	NM_009844	CTGGGACTATCCATCCACCA	TGTCTCCGAGGAAACCTGAC
**EPOr**	BC_046282	CAACAGCGGACACATCGAGTT	TAGCGAGGAGAACCGGACG
**GATA-1**	NM_008089	TGGAATCCAGACGAGGAACC	CTCCAGCCAGATTCGACCC
**FOG-1**	NM_009569	TCCCCTGAGAGAGAAGAACCG	GCAGCATCCTTAGCCAGCA
**TAL-1**	NM_011527	CTAGGCAGTGGGTTCTTTGGG	CCTCTTCACCCGGTTGTTGTT

36B4 = acidic ribosomal phosphoprotein PO; HPRT = hypoxanthine guanine phosphoribosyl transferase; ABC = ATP-binding cassette transporter; SR-BI = scavenger receptor class B1; EPOr = erythropoietin receptor; GATA-1 = globin transcription factor 1; FOG-1 = friend of GATA-1; Tal-1 = basic helix-loop-helix factor.

Immunoblotting on protein from peritoneal macrophages was performed as described previously [28]. In short, after running equal amounts of total cell protein (60 µg) on a 7.5% SDS-PAGE gel, ABCA1 was detected using murine monoclonal ABCA1 primary antibody (AC-10; a kind gift from Dr. M. Hayden, Vancouver, Canada) and ABCG1 was detected using polyclonal rabbit anti-mouse ABCG1 primary antibody (Novus Biologicals, Cambridge, UK) and a peroxidase-conjugated goat-anti-mouse IgG secondary antibody (Jackson ImmunoResearch, Suffolk, UK). Murine monoclonal β-actin antibody (Santa Cruz Biotechnology, Heidelberg, Germany) was used as a loading control. Finally, immunolabeling was detected by enhanced chemiluminescence (Thermo Scientific, Rockford, USA).

### Data analyses

Data are expressed as mean ± S.E.M. To compare differences among groups One-way ANOVA with Tukey's post-test was performed using GraphPad Prism version 4.00 for Windows (GraphPad Software, San Diego, CA, USA, www.graphpad.com). A *P* value<0.05 was considered statistically significant.

## Results

### Effect of macrophage ABCA7 deficiency on plasma lipid levels, atherogenesis and macrophage ABCA1 expression

**Figure 1:**
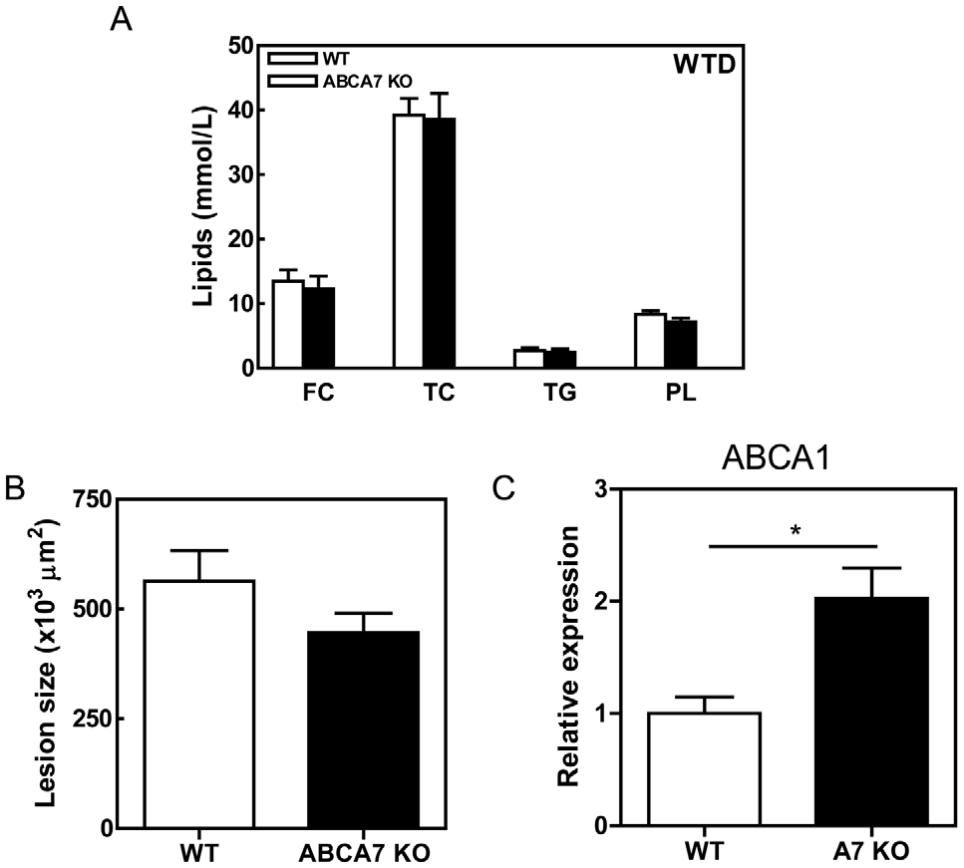
Effects of macrophage ABCA7 deficiency on plasma lipid levels, atherosclerosis and ABCA1 expression in peritoneal macrophages from LDLr KO mice reconstituted with WT and ABCA7 KO bone marrow. (**A**) Blood samples were drawn after an overnight fast. The concentrations of cholesterol, phospholipids and triglycerides in serum were determined using enzymatic colorimetric assays. (**B**) The mean lesion area (µm^2^) was calculated from Oil red O-stained cross-sections of the aortic root at the level of the tricuspid valves. (**C**) Guanidium thiocyanate-phenol was used to extract total RNA from cells and mRNA expression was determined by real-time RT-PCR. Hypoxanthine Guanine Phosphoribosyl Transferase (HPRT), β-actin, and acidic ribosomal phosphoprotein PO (36B4) were used as the standard housekeeping genes. Relative gene expression was calculated by subtracting the threshold cycle number (Ct) of the target gene from the average Ct of housekeeping genes and raising two to the power of this difference. Values represent the mean ± SEM of ≥6 mice per group. Statistically significant difference *p<0.05.

ABCA7 was selectively disrupted in hematopoietic cells, thus including macrophages, by transplantation of bone marrow from ABCA7 KO mice into LDLr KO mice, which represent a recognized model for the development of atherosclerosis. After a recovery period of 8 weeks on regular murine chow diet, the transplanted mice were challenged with a high cholesterol, high fat WTD containing 0.25% cholesterol and 15% cacao butter. No significant effect of macrophage ABCA7 deficiency was observed on serum free cholesterol (FC) levels, total cholesterol (TC), triglyceride (TG) or phospholipids (PL) (**Fig. 1A**). The effect on atherosclerosis susceptibility was analyzed in the aortic root of the transplanted LDLr KO mice after 10 weeks WTD feeding. Deletion of ABCA7 in macrophages did not significantly modify the lesion size (p>0.05) (**Fig. 1B**). Interestingly, the mRNA expression of the key lipid transporter ABCA1 was up-regulated 2-fold in ABCA7-deficient peritoneal macrophages (p = 0.028) (**Fig. 1C**). We have previously shown that macrophage ABCA1 overexpression inhibits atherosclerotic lesion progression in LDLr KO mice [29]. Thus, to gain further insight into the impact of ABCA7 expression on lipid metabolism and atherogenesis, the effect of combined macrophage ABCA1 and ABCA7 disruption was evaluated by means of BMT.

### Effect of combined macrophage ABCA1 and ABCA7 disruption on plasma lipids levels in LDLr KO mice

**Figure 2:**
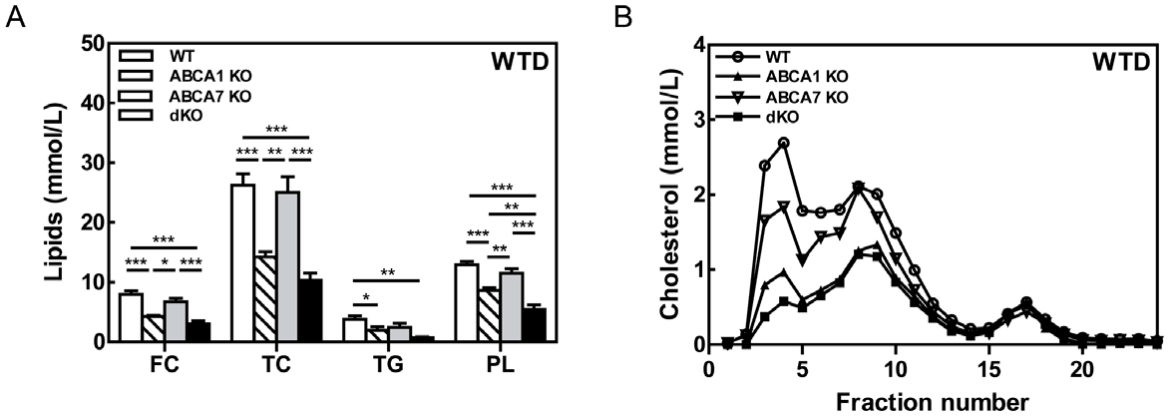
Plasma lipid levels and cholesterol lipoprotein distribution profile after 10 weeks WTD feeding of LDLr KO mice reconstituted with WT, ABCA1 KO, ABCA7 KO and dKO bone marrow. Blood samples were drawn after an overnight fast. (**A**) The concentrations of cholesterol, phospholipids and triglycerides in serum were determined using enzymatic colorimetric assays. (**B**) The distribution of cholesterol over the different lipoproteins was determined by fractionation of serum from individual mice using a Superose 6 column. Fractions 2 to 5 represent VLDL; fraction 6 to 14, LDL; and fractions 15 to 20, HDL, respectively. Values represent the mean±SEM of ≥6 mice per group. Statistically significant difference *p<0.05, **p<0.01, and *** p<0.001.

Bone marrow from WT, ABCA1 KO, ABCA7 KO, and dKO mice was transplanted into LDLr KO mice. From 8 weeks after bone marrow transplantation, the transplanted mice were challenged with the WTD to induce atherosclerotic lesion development. Similar as in the first BMT experiment, ABCA7 deletion in bone marrow-derived cells did not affect serum TC, TG or PL levels (**Fig. 2A**). However, in agreement with previous studies [30], deletion of ABCA1 in bone marrow cells resulted in significantly lower serum lipid levels compared to WT transplanted mice when fed WTD (TC: 0.5-fold; p<0.001, PL: 0.7-fold, p<0.001, TG: 0.5-fold, p<0.05). A similar decrease in serum lipids levels was found in LDLr KO mice transplanted with bone marrow lacking both ABCA1 and ABCA7 (TC: 0.4-fold; p<0.001, PL: 0.4-fold, p<0.001, TG: 0.2-fold; p<0.01).

Fractionation of serum lipoproteins showed that ABCA1 and dKO transplanted mice exhibited lower VLDL and LDL cholesterol levels (**Fig. 2B** **and** **Table 2**). VLDL production analysis showed a significantly lower VLDL production rate in mice lacking ABCA1 in bone-marrow derived cells (1.8-fold; p<0.05) compared to control, which is in line with the observed reduction in plasma TG levels in LDLr KO mice transplanted with ABCA1 or ABCA1/A7 deficient bone marrow cells. No significant effects of macrophage ABCA1, ABCA7 or combined ABCA1/ABCA7 deficiency were observed on HDL cholesterol levels.

**Table 2:** Serum cholesterol levels in WT, ABCA1 KO, ABCA7 KO and dKO bone marrow transplanted mice on chow and WTD.

Mice	Time(wks)	Diet	VLDL-C(mmol/L)	LDL-C(mmol/L)	HDL-C(mmol/L)
**WT**	8	Chow	0.50±0.04	3.6±0.08	2.9±0.18
**ABCA1 KO**	8	Chow	0.45±0.09	3.2±0.05^*^	2.9±0.16
**ABCA7 KO**	8	Chow	0.73±0.10	3.9±0.19	2.9±0.19
**dKO**	8	Chow	0.45±0.03	3.8±0.11	2.8±0.18
**WT**	18	WTD	7.0±1.7	11.2±1.1	1.8±0.26
**ABCA1 KO**	18	WTD	2.4±0.8^*^	6.4±0.9^*^	1.5±0.41
**ABCA7 KO**	18	WTD	4.7±1.4	9.4±1.1	1.6±0.40
**dKO**	18	WTD	1.4±0.3^*^	5.9±1.2^*^	1.5±0.28

Serum cholesterol levels were measured in LDLr^−/−^ at 8 and 18 weeks after transplantation with bone marrow from WT, ABCA1 KO, ABCA7, or dKO mice. At 8 weeks after bone marrow transplantation, the regular chow diet was switched to a high-cholesterol diet. Data represent the means±SEM of 12–16 mice. Statistical significance of *p<0.05, **p<0.01, and ***p<0.001 compared with WT transplanted mice. Abbreviations: WTD = Western-type diet; VLDL = very-low-density lipoprotein; LDL = low-density lipoprotein; HDL = high-density lipoprotein; C = cholesterol.

### Effect of macrophage ABCA1 and ABCA7 disruption on foam cell formation and atherosclerotic lesion formation

**Figure 3:**
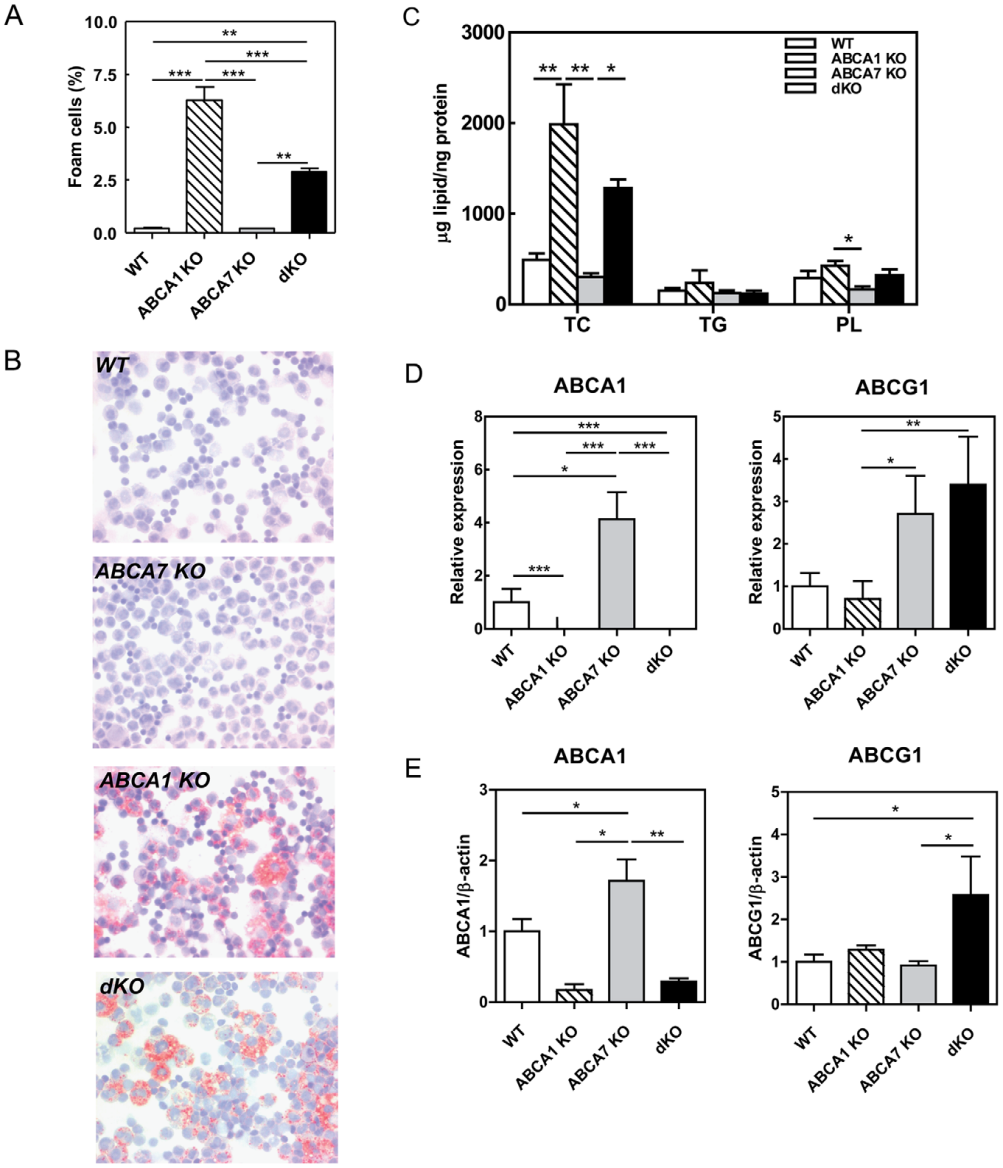
Effect of macrophage ABCA1, ABCA7, and combined ABCA1/ABCA7 deficiency on *in vivo* foam cell formation, and mRNA and protein expression levels of ABCA1 and ABCG1. (**A**) Peritoneal leukocytes were analyzed using a hematology Sysmex XT-2000iV analyzer and the number of macrophage foam cells were quantified as percentage of the total amount of isolated cells. Values represent the mean±SEM of ≥5 mice per group. Statistically significant difference **p<0.01, and ***p<0.001 vs. WT. (**B**) Photomicrographs of oil-red O-stained cytospins of peritoneal cells. Original magnification ×400. (**C**) Lipids were extracted from peritoneal leukocytes with organic solvents. (**D**) Guanidium thiocyanate-phenol was used to extract total RNA from peritoneal macrophages and mRNA expression was determined by real-time RT-PCR. Hypoxanthine Guanine Phosphoribosyl Transferase (HPRT), β-actin, and acidic ribosomal phosphoprotein PO (36B4) were used as the standard housekeeping genes. Relative gene expression was calculated by subtracting the threshold cycle number (Ct) of the target gene from the average Ct of housekeeping genes and raising two to the power of this difference. WT samples were all calibrated to an arbitrary unit. (**E**) Western-blot analyses were performed to quantify the protein expression levels of ABCA1 and ABCG1 in peritoneal leukocytes. Values represent the mean ± SEM of 4–6 mice per group. Statistically significant difference *p<0.05, **p<0.01 and ***p<0.001.

As *in vivo* foam cell formation within the peritoneal cavity is indicative for the susceptibility to atherosclerotic lesion development [31], [32], the effect of deletion of macrophage ABCA1 and/or ABCA7 on the presence of foam cells in the peritoneal cavity was investigated. Disruption of macrophage ABCA7 did not induce foam cell accumulation in the peritoneal cavity (0.20±0.03% [n = 5] compared to 0.20±0.07% [n = 7] for control animals; p>0.05) (**Fig. 3A**). As expected, a 30-fold increase in foam cell formation was detected in ABCA1 KO transplanted mice as compared to the controls (6.25±0.65% [n = 8] vs 0.20±0.07% [n = 7]; p<0.001). DKO transplanted animals exhibited also a highly significant increase in the amount of foam cells compared to control animals (2.88±0.19% [n = 5] compared to WT 0.20±0.07% [n = 7]; p<0.01), which, however, was less severe than in single ABCA1 KO transplanted mice (p<0.001) (**Fig. 3A**). The increase in foam cells in ABCA1 KO and dKO transplanted mice was also indicated by microscopic visualization using Oil red O-stained cytospins (**Fig. 3B**) and confirmed by extracting tissue lipids with organic solvents (**Fig. 3C**). Lipid extraction analyses from cells showed an increase in TC levels in ABCA1 KO and dKO transplanted mice compared to WT (4-fold, p<0.01 and 2.6-fold, p>0.05, respectively) transplanted mice (**Fig. 3C**).

**Figure 4:**
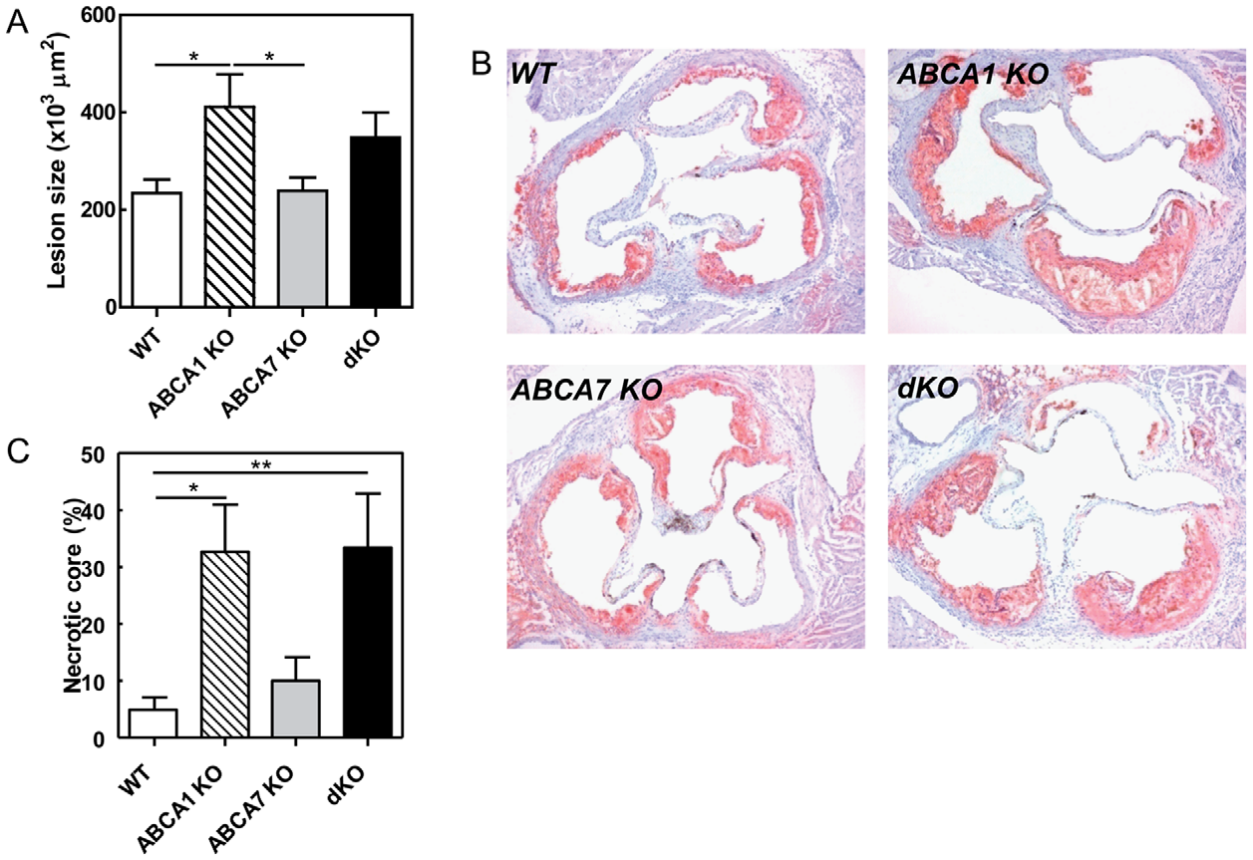
Atherosclerosis in the aortic root of LDLr KO mice reconstituted with WT, ABCA1 KO, ABCA7 KO and dKO bone marrow. (**A**) The mean lesion area (µm^2^) was calculated from Oil red O-stained cross-sections of the aortic root at the level of the tricuspid valves. (**B**) Representative pictures are shown. (**C**) The percentage of necrotic core of the total lesion was analysed using TUNEL staining. Values represent the mean±SEM of ≥6 mice per group. Statistically significant difference *p<0.05.

To investigate the effect of deletion of macrophage ABCA1 and/or ABCA7 on atherosclerotic lesion development, lesion sizes in Oil red-O stained sections of the aortic root were quantified after 10 weeks WTD feeding. Again, single deletion of macrophage ABCA7 did not affect atherosclerotic lesion development as compared to control animals (239±27×10^3^ µm^2^ [n = 10] compared to 234±28×10^3^ µm^2^ [n = 13]; p>0.05) (**Fig. 4A and B**). Furthermore, in agreement with our previous study [30], single disruption of macrophage ABCA1 resulted in a 1.8-fold increase in atherosclerotic lesion size as compared to lesions in WT transplanted animals (412±67×10^3^ µm^2^ [n = 11] compared to 234±28×10^3^ µm^2^ [n = 13]; p<0.05)(**Fig. 4**). Combined deletion of macrophage ABCA1 and ABCA7 led to a 1.3-fold increase in atherosclerotic lesion, which was however not statistically significant different from the WT transplanted mice (348±52×10^3^ µm^2^ [n = 7] compared to 234±28×10^3^ µm^2^ [n = 13]). The percentage of TUNEL-positive nuclei did not differ between experimental groups (WT: 3.5±1.3%, ABCA1 KO: 8.3±2.5%, ABCA7 KO: 6.8±2.6%, dKO: 2.3±0.4); however, both ABCA1 KO and dKO transplanted mice showed a significant increase in necrotic core compared with control animals (33±8 and 33±9% of total lesion compared with 5±2% of total lesion, p<0.05 and p<0.01, respectively) (**Fig. 4C**). Lesions were mainly occupied by macrophages as shown by F4/80 staining (**Figure S1**).

Interestingly, dKO transplanted mice, thus, showed a less severe increase in both foam cells and atherosclerotic lesion size compared with ABCA1 KO transplanted mice, indicating that ABCA7 deletion attenuates the effect of ABCA1 deletion on foam cell formation and atherosclerosis. To determine what might be causitive for the attenuated macrophage foam cell formation *in vivo* upon combined deletion of ABCA1 and ABCA7, the expression of ABCG1 was determined in the peritoneal cells as well as ABCA1 and ABCA7. Absence of ABCA1 in macrophages did not affect the mRNA expression levels of ABCG1 (**Fig. 3D**) nor ABCA7 (data not shown). Similarly to the findings from the first BMT study (**Fig. 1C**), macrophages of ABCA7 KO transplanted mice showed a 5-fold significant increase in ABCA1 mRNA expression compared to control animals (p<0.05). In agreement, western-blot analyses showed that also ABCA1 protein expression levels were upregulated (1.7-fold, p<0.05) in macrophages deficient in ABCA7 (**Fig. 3E**). As expected, deletion of macrophage ABCA1 resulted in a complete absence of ABCA1 mRNA and thus abrogated the upregulation of ABCA1 upon deletion and ABCA7. Importantly, also the mRNA expression levels of ABCG1 were increased in ABCA7 KO and dKO transplanted mice compared to control and ABCA1 KO transplanted mice, which, however, only reached significance compared to ABCA1 KO transplanted mice (p<0.05 and p<0.01, respectively). However, protein expression levels of ABCG1 were only induced in macrophages with combined deletion of ABCA1 and ABCA7 (2.5-fold, p<0.05) (**Fig. 3E**). These data suggest that the decreased foam cell formation in dKO transplanted animals as compared to single ABCA1 KO transplanted mice might be the consequence of enhanced efflux to HDL by ABCG1.

**Figure 5:**
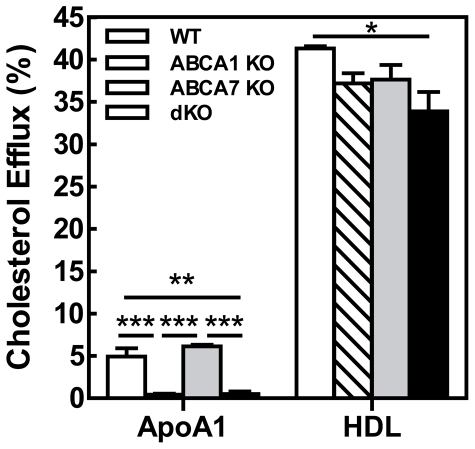
Effect of macrophage ABCA1, ABCA7, and combined ABCA1/ABCA7 deficiency on cellular cholesterol efflux. Thioglycollate-elicited peritoneal macrophages were isolated from male recipients at 18 weeks posttransplant. Cholesterol efflux from ^3^H-cholesterol-labeled cells incubated with DMEM/0.2% BSA alone or supplemented with either 10 µg/mL apoA-I or 50 µg/mL human HDL was determined after 24 hours of incubation. Results are the mean±SEM of 4 mice per group. Statistically significant difference *p<0.05.

Therefore, next the effect of ABCA1 and ABCA7 expression on the efflux of cholesterol from peritoneal macrophages to apoA-I and HDL was determined *in vitro*. The percentage of cholesterol effluxed from WT cells and from cells lacking ABCA7 to either apoA-I or HDL did not differ, whereas deletion of ABCA1 or combined deletion of ABCA1 and ABCA7 led to a completely abolished efflux to apoA-I (both≥95% decrease p<0.001 and p<0.001, respectively) (**Fig. 5**). Importantly, the absence of both ABCA1 and ABCA7 resulted in a moderate but significant 18% reduction in efflux to HDL compared to control cells (33.9±1.6% compared to 41.3±0.3%; p<0.05). The anticipated increase in cholesterol efflux to HDL could, thus, not be confirmed in peritoneal macrophages in culture.

### Disturbed spleen morphology in combined ABCA1 and ABCA7-deficient bone marrow transplanted mice

**Figure 6:**
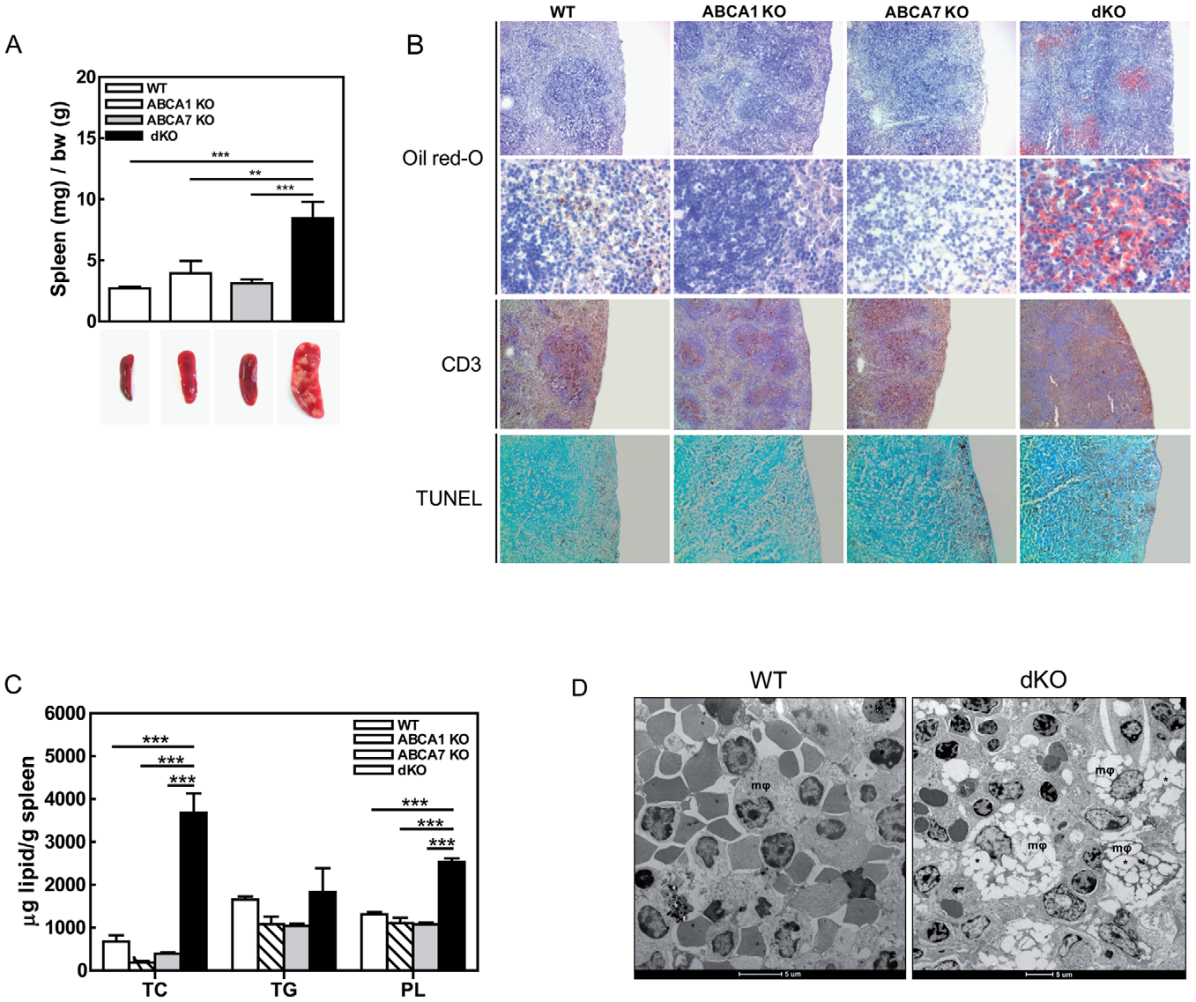
Effects of single macrophage ABCA7 and combined macrophage ABCA1 and ABCA7 deficiency on spleen. (**A**) Relative spleen weights of transplanted mice to body weight (bw) and representative images are shown. (**B**) Cryosections of spleens from all groups of mice were stained for lipid accumulation using Oil-red O staining (original magnification ×50 for first row and ×400 for second row), against lymphocyte CD3 antigen by immunohistochemistry (original magnification ×40) and for apoptosis using TUNEL staining (original magnification ×40). (**C**) Lipids were extracted from spleens with organic solvents. Values represent the mean±SEM of ≥9 mice per group. Statistically significant difference **p<0.01 and ***p<0.001. (**D**) Electron microscopic images of spleens from WT and dKO mice. Macrophages (mφ) of dKO transplanted mice exhibited massive cytoplasmic lipid accumulation (*).

Both on chow and on WTD, WT, ABCA1 KO, ABCA7 KO, and dKO transplanted mice did not differ in body weight (data not shown). Furthermore, no differences in relative weight, lipid content and cell composition in liver, thymus, and lymph nodes were observed (data not shown). Interestingly, the relatively spleen weight of dKO transplanted mice was increased 3-fold (8.44±1.35 mg/g body weight (bw) compared with 2.70±0.14 mg/g bw in control mice; p<0.001) (**Fig. 6A**), while spleen weights of ABCA1 KO and ABCA7 KO transplanted mice did not differ compared to WT transplanted mice. In addition, macroscopically white foci were visible in spleens of dKO transplanted mice (**Fig. 6A**), which were consistent with lipid accumulation, visualized by Oil red-O staining of cryostat sections of the spleen (**Fig. 6B**). In agreement, lipid extraction analyses from the spleen showed a significant increase in TC and PL levels in dKO transplanted mice compared to WT (TC: 5.4-fold, p<0.001; PL: 1.9-fold, p<0.001), ABCA1 KO (TC: 19.7-fold, p<0.001; PL: 2.3-fold, p<0.001), and ABCA7 KO (TC: 9.4-fold, p<0.001; PL: 2.4-fold, p<0.001) transplanted mice (**Fig. 6C**). Electron microscopic analyses were performed to determine in which cell type and cellular compartment the accumulation of excessive amounts of lipid occured. Interestingly, massive cytoplasmic lipid accumulation was observed in the macrophages of dKO transplanted mice, which was not observed in spleens of WT transplanted mice (**Fig. 6D**).

Interestingly, the spleen of ABCA7 KO and dKO transplanted LDLr KO mice also showed an increase in apoptotic cells (**Fig. 6B**), which might be caused by the impaired phagocytic capacity in ABCA7 KO transplanted mice.

**Figure 7:**
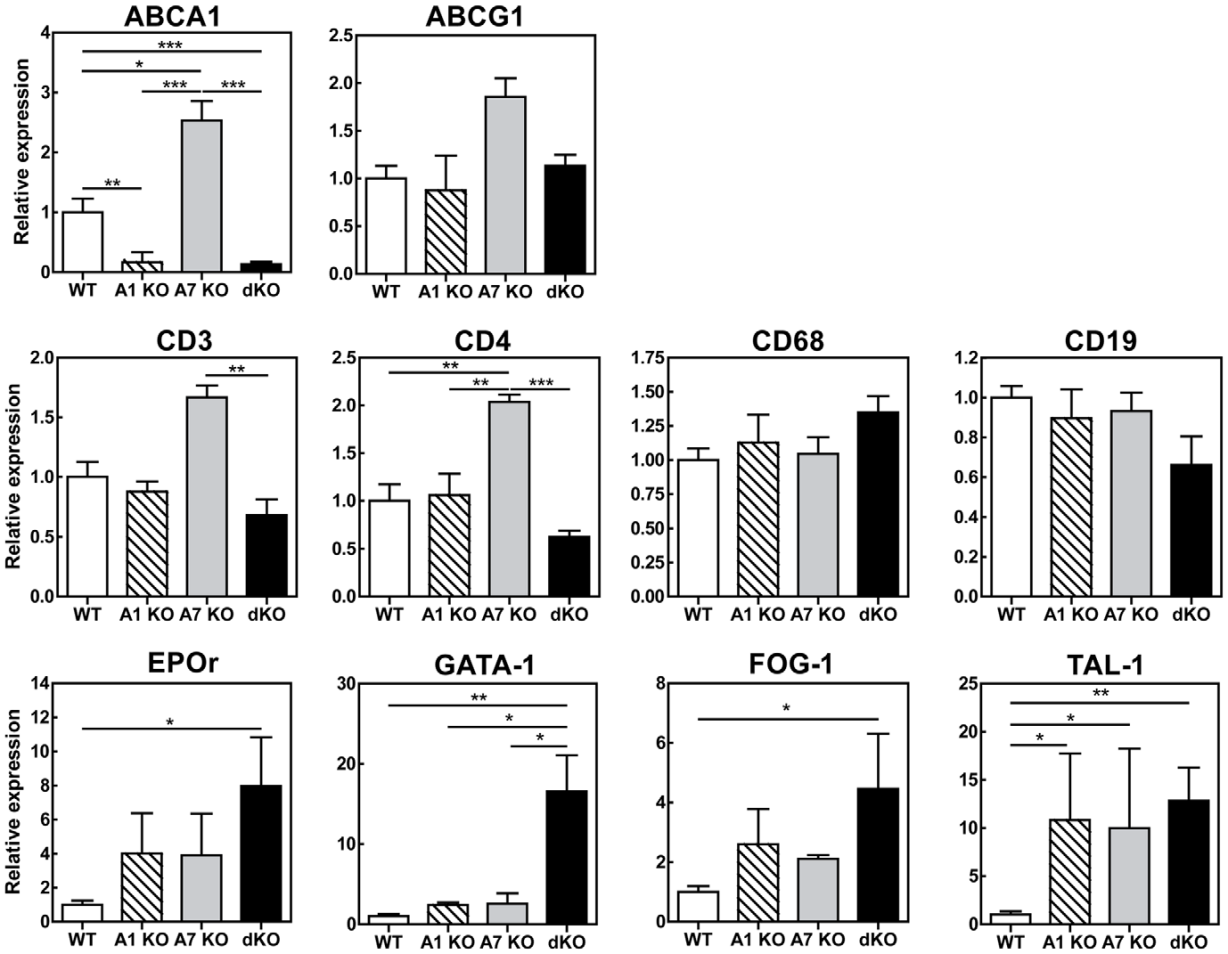
mRNA expression levels of genes of interest in spleens from LDLr KO mice reconstituted with WT, ABCA1 KO, ABCA7 KO and dKO bone marrow. Guanidium thiocyanate-phenol was used to extract total RNA from organs and mRNA expression was determined by real-time RT-PCR. Hypoxanthine Guanine Phosphoribosyl Transferase (HPRT), β-actin, and acidic ribosomal phosphoprotein PO (36B4) were used as the standard housekeeping genes. Relative gene expression was calculated by subtracting the threshold cycle number (Ct) of the target gene from the average Ct of housekeeping genes and raising two to the power of this difference. WT samples were all calibrated to an arbitrary unit. Values represent the mean ± SEM of 4–6 mice per group. Statistically significant difference *p<0.05, **p<0.01 and ***p<0.001.

To further define the effect of combined deletion of ABCA1 and ABCA7 on splenic function, mRNA analyses were performed (**Fig. 7**). Similar as observed for peritoneal macrophages, mRNA expression of ABCA1 was increased in the spleen of ABCA7 KO transplanted mice compared to controls (p<0.05). This increase was not due to an increase in macrophages in spleens of ABCA7 KO transplanted mice, as no difference in CD68 mRNA expression was observed between the 4 groups. As expected mRNA expression of ABCA1 was diminished in spleens of ABCA1 KO and dKO transplanted mice (p<0.01 and p<0.0001, respectively). In contrast to the findings in peritoneal macrophages, no difference was observed in expression of ABCG1 in the spleen. Furthermore, deletion of ABCA1 did not affect ABCA7 mRNA expression (data not shown).

**Figure 8:**
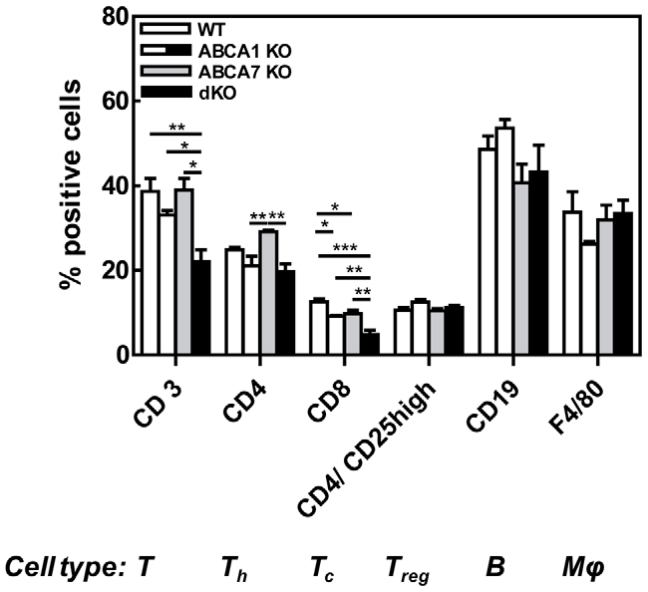
Effects of single macrophage ABCA7 and combined macrophage ABCA1 and ABCA7 deficiency on cellular spleen composition. Spleen cell subsets measured by flow cytometry expressed as percentage of positive cells. Values represent the mean ± SEM of 5 mice per group. Statistically significant difference *p<0.05, **p<0.01 and ***p<0.001.

Cell subsets composition of the spleen was evaluated by determing mRNA expression of CD3, CD4, CD68, and CD19 (Fig. 7). ABCA7 KO transplanted mice exhibited increased splenic mRNA expression in T helper cells (CD4) compared to controls (p<0.01). No differences were observed in B cells (CD19) and macrophages (CD68) between the groups. Subsequently, the effect of combined deletion of ABCA1 and ABCA7 on splenic cell population was examined by FACS analysis (**Fig. 8**). Deletion of ABCA1 and/or ABCA7 did not affect splenic F4/80^+^ macrophages, CD19^+^ B cells, and CD4^+^/CD25^high^ regulatory T cells compared to control mice. Single ABCA7 deficiency did show an increase in CD4^+^ T helper cells, which, however, failed to reach significance (29±0.4% compared with 25±0.5% in WT transplanted animals; p>0.05). This increase in CD4^+^ cells was in line with the increase in CD4 mRNA expression measured by qPCR analysis (**Fig. 7**). Remarkably, disruption of both ABCA1 and ABCA7 on hematopoietic cells resulted in a decrease in the percentage of splenic CD3^+^ T cells (22±3% compared with 39±4% in WT transplanted animals; p<0.01) (**Fig. 8**), which is the result of a trend towards a reduction in T helper cells (CD4^+^)(p>0.05) and a significant reduction in cytotoxic T cells (CD8^+^)(p<0.001). A decrease in splenic CD3^+^ T cells in dKO transplanted mice was also indicated by immunohistochemical staining against lymphocyte CD3 antigen (**Fig. 6B**). WT, ABCA1 KO, and ABCA7 KO transplanted mice showed strong CD3^+^ staining in the centre of white pulp areas of the spleen, which represents the periarterial lymphatic sheath (PALS). DKO transplanted mice, however, showed a disturbed spleen morphology, as the white and red pulp areas were difficult to distinguish from each other and no strong CD3^+^ staining could be observed, even not in the PALS. No effect was observed on lymphocyte counts in the circulation nor in the thymus and lymph nodes (data not shown), indicating that the observed effects on T cells are specific for spleen.

F4/80 staining showed, as expected, distinct macrophage-rich areas constituting the red pulp of spleens of the WT, ABCA1 KO, and ABCA7 KO transplanted mice (**Fig S2**). However, the morphology of the spleen of dKO transplanted mice was disrupted, as the white and red pulp areas were difficult to distinguish from each other. F4/80 staining of spleens of dKO transplanted mice showed macrophages distributed throughout the spleen and not localized in specific areas. As the percentage of macrophages and B cells in the spleens of the dKO transplanted mice was not changed (**Fig. 8**), it is likely that the increase in spleen size was due to more (lipid-filled) macrophages, as shown by Oil red-O staining (**Fig. 6B**) and electron microscopy (**Fig. 6D**).

To evaluate whether the increase in spleen weight of dKO transplanted mice was also a direct result of increased erythropoiesis, mRNA gene expression of different transcriptors involved in splenic erythropoiesis was determined: ea erythropoietin receptor (EPOr), globin transcription factor 1 (GATA-1), friend of GATA-1 (FOG-1), and basic helix-loop-helix factor TAL-1. The expression of EPOr, involved in the proliferation and differentiation of the colony-forming units-erythrocyte (CFU-E) and proerythroblasts, was significantly increased in dKO transplanted animals (p<0.05) (**Fig. 7**). In addition, dKO transplanted mice exhibited a significant increase in GATA-1 (p<0.01) and FOG-1 (p<0.05) genes, involved in the differentiation of committed erythroid progenitor cells. Furthermore, disruption of both ABCA1 and ABCA7 in hematopoietic cells resulted in a significant increase in TAL-1 expression (p<0.05), a gene involved in the commitment of hematopoietic cells to the erythroid lineage. Therefore, these data indicate that the expression of key regulators in erythropoiesis was affected in dKO transplanted animals. Accordingly, darker staining for erythroid lineage cells (TER 119) was observed in dKO transplanted animals, while no difference between groups was found in the cell proliferation rate (Ki67) (**Figure S2**). However, no differences in circulating red blood cells were observed between the four groups (data not shown).

## Discussion

ABCA7 is an ABC-transporter which shows the highest protein sequence homology with ABCA1, a key regulator of macrophage cholesterol and phospholipid efflux to lipid-poor apoA-I and other lipid-poor apolipoproteins [14]. Therefore, it was assumed that ABCA7 may also function in cellular lipid or cholesterol metabolism. In the current study, we, for the first time, show that specific hematopoietic ABCA7 deficiency does not affect WTD diet-induced atherosclerotic lesion development in LDLr KO mice. Noteworthy, ABCA1 expression was upregulated 2-fold in peritoneal macrophages lacking functional ABCA7, suggesting that crosstalk between ABC transporter family members seems feasible and that ABCA7, like other ABC transporters, may be part of a complex machinery controling lipid homeostasis in macrophages with possible (partly) overlapping functions. In agreement, ABCA2 (Calpe-Berdiel et al. unpublished), ABCA5 [25], and ABCG1 [33] deletion independently led to compensatory up-regulation of ABCA1 in these cells. In addition, our group previously demonstrated that LDLr KO mice overexpressing macrophage ABCA1 showed 3-fold smaller atherosclerotic lesions [29]. Increased macrophage ABCA1 expression might thus counteract the effect of ABCA7 deletion in foam cell formation and atherosclerosis. To gain further insight into the impact of ABCA7 expression on lipid metabolism and atherogenesis and to analyze potential synergistic properties, we next evaluated the effect of combined macrophage ABCA1 and ABCA7 disruption. After 10 weeks of WTD feeding, macrophage ABCA7 deficiency did not alter serum lipid or lipoprotein levels compared to control mice, which is in line with the results found in total body ABCA7-deficient mice reported by Kim *et al.* [24]. In line with previous findings [30], disruption of macrophage ABCA1 resulted in a decrease in lipid levels, which was similar to the reduction in lipid levels observed in mice with combined macrophage ABCA1 and ABCA7 deletion, indicating that this reduction is most likely attributable to the deficiency in macrophage ABCA1. We now show that this is, at least partly, due to decreased VLDL production by the liver upon disruption of ABCA1 in bone marrow-derived cells.

Despite the decreased serum lipid levels in ABCA1 KO and ABCA1/ABCA7 dKO transplanted mice, significantly higher amounts of foam cells were observed in the peritoneal cavity of these animals compared to controls, while also the susceptibility to atherosclerosis was increased. Single macrophage ABCA7 deficiency did not affect foam cell formation nor atherosclerotic lesion size. The observed increase in lesion size in dKO transplanted mice is, thus, most likely attributable to the deficiency in macrophage ABCA1 only. Interestingly, foam cell formation and susceptibility to atherosclerotic lesion development in dKO transplanted LDLr KO mice was less severe compared to single ABCA1 KO transplanted mice, indicating that the presence of ABCA7 augments macrophage foam cell formation and possibly atherosclerosis in the absence of ABCA1. However, it is important to note that dKO transplanted LDLr KO mice exhibited lower VLDL cholesterol levels compared to ABCA1 KO transplanted mice (0.6-fold compared with ABCA1 KO, p>0.05). Although the difference was not significant, prolonged exposure to lower VLDL cholesterol levels might have contributed to the observed reduction in atherosclerotic lesion development in the dKO transplanted mice. Macrophage apoptosis is an important feature of atherosclerotic plaque development and occurs during all stages of atherosclerosis with increasing frequencies as the plaque develops. Research directed at understanding the functional consequences of macrophage death in atherosclerosis has revealed opposing roles for apoptosis in atherosclerotic plaque progression [34], [35]. ABCA7 has been suggested to play a role in phagocytosis of apoptotic cells [17], [36]. Therefore, in addition to the anticipated role of ABCA7 in cholesterol homeostasis and cholesterol efflux, ABCA7 may also affect atherosclerosis by its role in phagocytosis, which can be pro- or antiatherogenic. However, no differences in atherosclerotic lesion formation was observed in ABCA7 KO mice compared to controls. Still, a positive correlation between the amount of foam cells and the susceptibility to atherosclerosis was found, indicating that atherogenesis, under the conditions studied, is likely to occur independently of an anticipated role for ABCA7 in phagocytosis.

Lipid accumulation in cells, leading to the formation of foam cells, might be due to a defect in the efflux of lipids out of the cells to exogenous lipid acceptors. ABCA1 and ABCA7 have a high degree of sequence homology, including the first and fourth extracellular loops [22], suggesting that ABCA7 might be able to bind apolipoproteins and mediate cellular lipid efflux. Wang *et al*. [22] previously reported, that HEK293 cells expressing murine ABCA7 displayed increased apoA-I binding. These findings indicate that ABCA7 shares the functional properties of ABCA1 to transport phopholipids and cholesterol in response to stimulation with apoA-I. However, the ability and magnitude of the lipid transport activity of ABCA7 is disputed [20], [21], [22], [24]. HEK293 cells expressing human ABCA7 demonstrated apoA-I-mediated release of both phospholipids and cholesterol [20], [21]. Wang *et al.* [22] have shown that mouse ABCA7 only promotes apoA-I-dependent phospholipid release, but not cholesterol. In contrary, Kim *et al.* [24] showed that macrophages of ABCA7-deficient mice displayed a normal capacity to efflux both phosphatidylcholine and cholesterol to apoA-I. The lack of an effect on macrophage lipid efflux was postulated to be the consequence of the absence of ABCA7 from the plasma membrane of murine macrophages, so that no direct physical interaction with apoA-I is possible. Ikeda *et al*. [15] reported that the human type I ABCA7 (full length cDNA) is localized on the plasma membrane and mediates release of cholesterol and phospholipids to apoA-I, while the human type II (spliced variant cDNA) is primarily localized in the endoplasmic reticulum and might be involved in intracellular lipid trafficking. In line with these findings, several other studies [17], [23], [24] demonstrated that ABCA7 does not function to export lipid from cells, but rather plays an intracellular role. In the present study, we confirm that single disruption of ABCA7 in mouse peritoneal macrophages does not affect cholesterol efflux to apoA-I, suggesting that ABCA7 has no cholesterol efflux functionality in this cell type. We, however, cannot exclude that the loss of function of ABCA7 is compensated by ABCA1. As earlier mentioned, mRNA expression and protein levels of ABCA1 were significantly induced in peritoneal macrophages of ABCA7 KO transplanted mice, suggestive of a compensatory up-regulation of ABCA1 in absence of ABCA7. Disruption of ABCA1 or both ABCA1 and ABCA7 completely ablated cholesterol efflux to apoA-I, indicating that loss of ABCA1 function could not be rescued by compensatory mechanisms. In line with these findings, it has been previously reported that ABCA7 does not rescue the systemic phenotype induced by the *in vivo* loss of ABCA1 function in either mouse or man [37],[38],[39]. Efflux of cholesterol to HDL was only significantly reduced in peritoneal macrophages of dKO transplanted mice. Surprisingly, despite the observed reduction in cholesterol efflux *in vitro*, *in vivo* peritoneal foam cell formation was reduced upon combined deletion of ABCA1 and ABCA7. This is probably the consequence of the observed up-regulation of ABCG1 expression *in vivo* in cells lacking ABCA1 and ABCA7. Single deletion of ABCA1 did not affect ABCG1 protein expression. The observed up-regulation of ABCG1 in ABCA1/ABCG1 dKO macrophages is thus most likely a direct effect of ABCA7 deletion and not of the absence of ABCA1.

However, the reduced cholesterol efflux to HDL from peritoneal macrophages of dKO transplanted mice in culture seems to be in contrast to the observed compensatory upregulation of ABCG1 expression, which can be interpreted that the increase in ABCG1 cannot compensate for the absence of ABCA7.

Future generation of ABCA1/ABCG1/ABCA7 triple knockout mice is expected to unequivocally establish the importance of ABCA7 for macrophage lipid homeostasis and atherosclerotic lesion development.

The highest expression of ABCA7 in mouse can be found in myelo-lymphatic tissues, such as thymus, spleen, lymph node, and bone marrow and moderate expression in lung, brain, and adrenal glands [14], [22]. No differences in weight or morphology of the thymus, lymph nodes, and liver were observed between the four groups. Interestingly, severe splenomegaly was observed in dKO transplanted mice, whereas no such phenotype was present in WT, ABCA1 KO, and ABCA7 KO transplanted mice. The spleens of dKO transplanted mice contained white foci, which corresponded with extreme lipid accumulation, as indicated by Oil red O staining. Furthermore, the amount of T cells was significantly reduced in spleens of these transplanted mice. In light of the predominant expression of ABCA7 in macrophages and other lympho-myeloid cell types and its anticipated role in hematopoiesis [16], it is conceivable that ABCA7 is involved in processes associated with immune functions. The observed reduction in T cells in spleens of dKO transplanted mice might, thus, be a direct result of impaired lymphopoiesis in absence of ABCA7. The reduction in splenic T-cells, however, was only observed when both ABCA1 and ABCA7 transporters are disrupted in hematopoietic cells, as single ABCA1 deficiency did not affect splenic T-cells.

Since ABCA7 is highly expressed on erythrocytes [22] and enhanced splenic erythropoiesis is a common cause of increased spleen size, mRNA expression levels of genes involved in erythropoiesis and erythroid lineage differentiation were analysed. mRNA gene expression of different transcription factors involved in splenic erythropoiesis, as well as the intensity of TER 119 staining were increased in LDLr KO transplanted with dKO bone marrow. Increased erythropoiesis might be the result of disturbed morphology of the spleen of dKO transplanted mice, due to lipid accumulation or lymphopenia. Kam *et al*. [40] has previously shown that although some erythropoiesis occurs in the spleen under normal conditions, most splenic erythropoiesis occurs as a response to hematologic stress. On the other hand, ABCA7 shows potential binding sites for transcription factors with roles in hematopoiesis [41]. Therefore, the effect on erythropoiesis observed in spleens of dKO transplanted mice might be thus a direct result of the hematopoietic ABCA7 deficiency.

In conclusion, single deletion of macrophage ABCA7 does not affect cellular cholesterol efflux and atherosclerotic lesion development in LDLr KO mice, possibly due to compensatory up-regulation of ABCA1 expression. In addition, no synergistic role of the transporters ABCA1 and ABCA7 in atherosclerotic lesion development was demonstrated, despite the anticipated roles of both transporters in macrophage lipid efflux and phagocytosis. Since dKO transplanted mice exhibited severe splenomegaly, associated with a decrease in CD3+ T cells and increased markers of erythropoiesis, ABCA7 might have a role in hematopoiesis in spleen.

## Supporting Information

Figure S1 **Effect of macrophage ABCA1, ABCA7, and combined ABCA1/ABCA7 deficiency on macrophage staining in atherosclerotic lesions.** Cryosections of the aortic root at the level of the tricuspid valves from all groups of mice were stained for macrophage F4/80 specific antigen. Original magnification ×400.
(EPS)

Figure S2 **Effects of single macrophage, ABCA1, ABCA7 and combined macrophage ABCA1 and ABCA7 deficiency on splenic macrophage, proliferation and erythroid cells.** Cryosections of spleens from all groups of mice were stained for macrophage F4/80 specific antigen, Ki67 cell proliferation rate marker and TER 119 erythroid lineage marker. Original magnification ×400.
(EPS)
