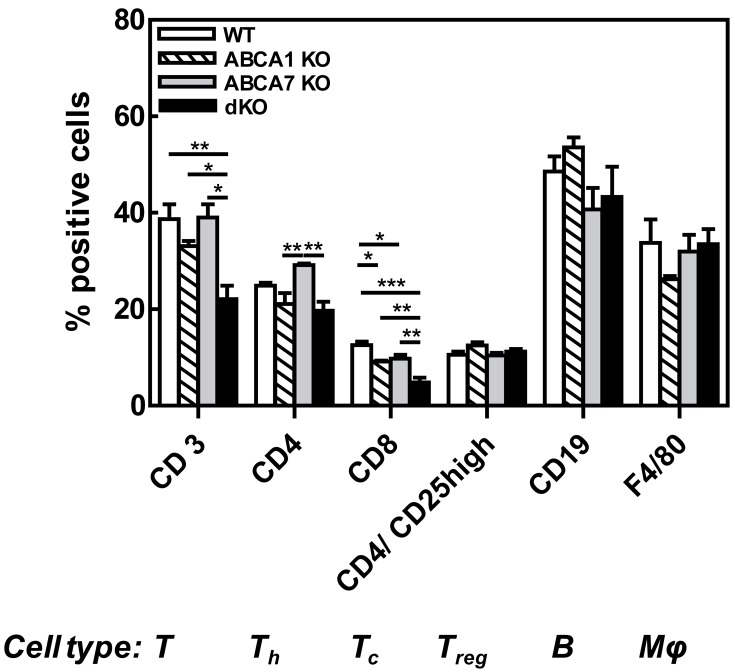# Correction: Effects of Deletion of Macrophage ABCA7 on Lipid Metabolism and the Development of Atherosclerosis in the Presence and Absence of ABCA1

**DOI:** 10.1371/annotation/577d9009-baf4-46ea-b44e-eda094b604db

**Published:** 2012-05-24

**Authors:** Illiana Meurs, Laura Calpe-Berdiel, Kim L. L. Habets, Ying Zhao, Suzanne J. A. Korporaal, A. Mieke Mommaas, Emmanuelle Josselin, Reeni B. Hildebrand, Dan Ye, Ruud Out, Johan Kuiper, Theo J. C. Van Berkel, Giovanna Chimini, Miranda Van Eck

There were errors in Figures 1-8. The correct Figures can be viewed here:

Figure 1 

**Figure:**
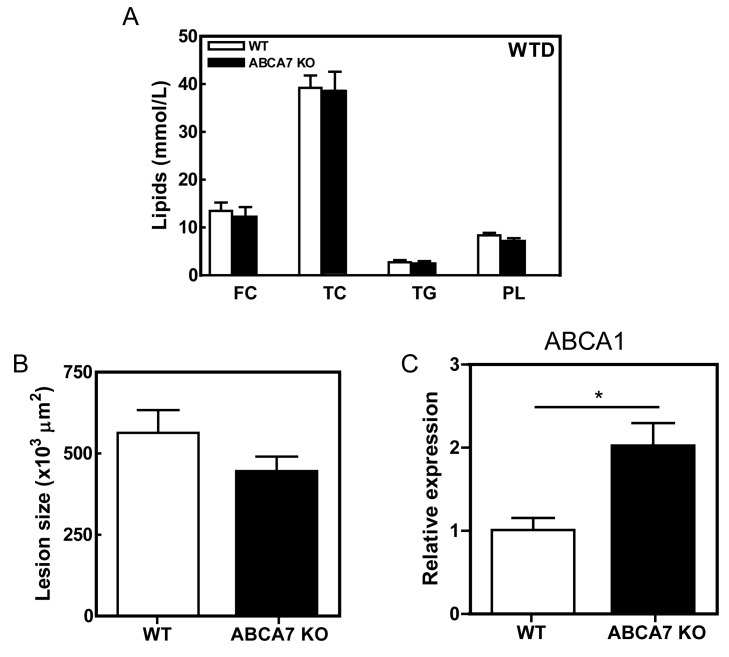


Figure 2 

**Figure:**
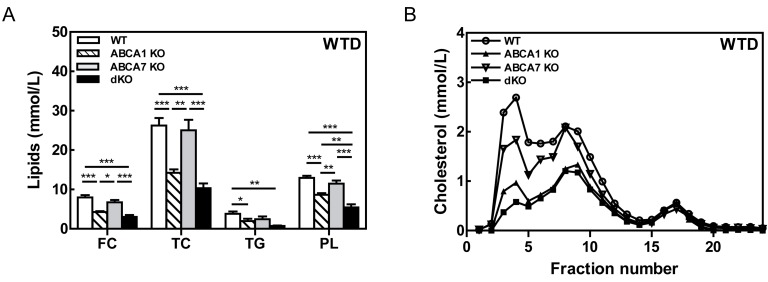


Figure 3 

**Figure:**
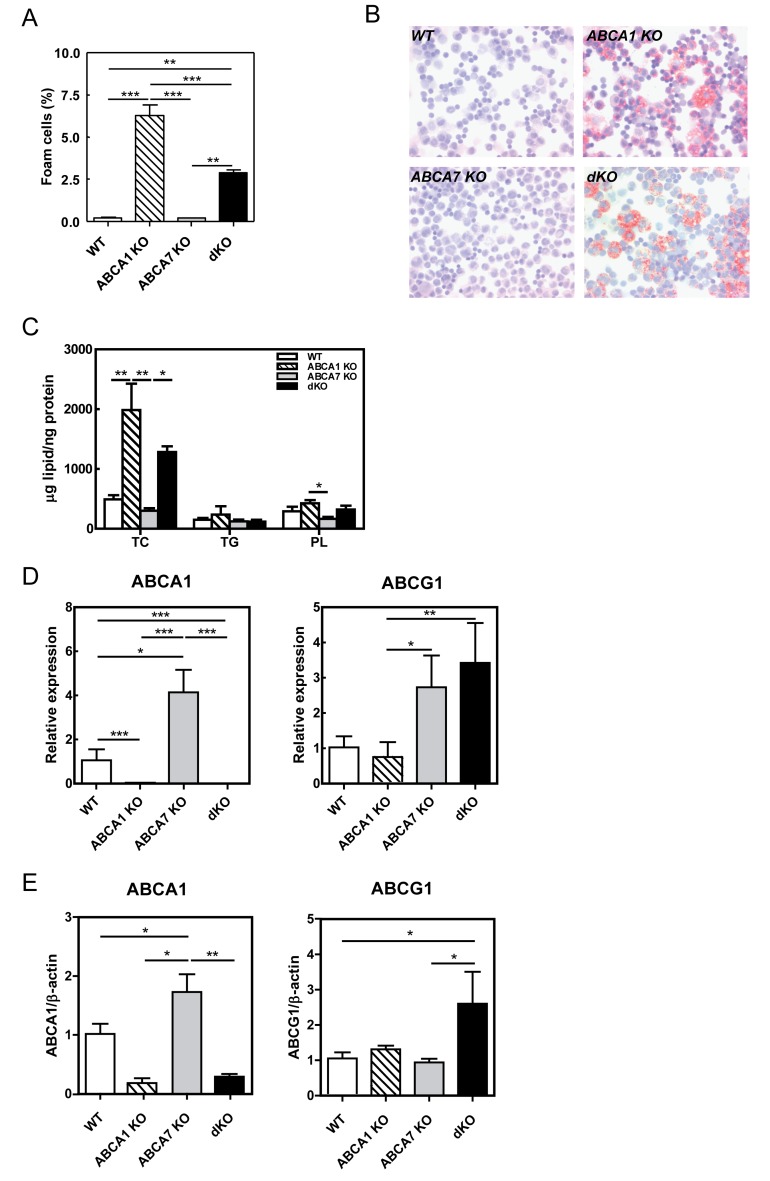


Figure 4 

**Figure:**
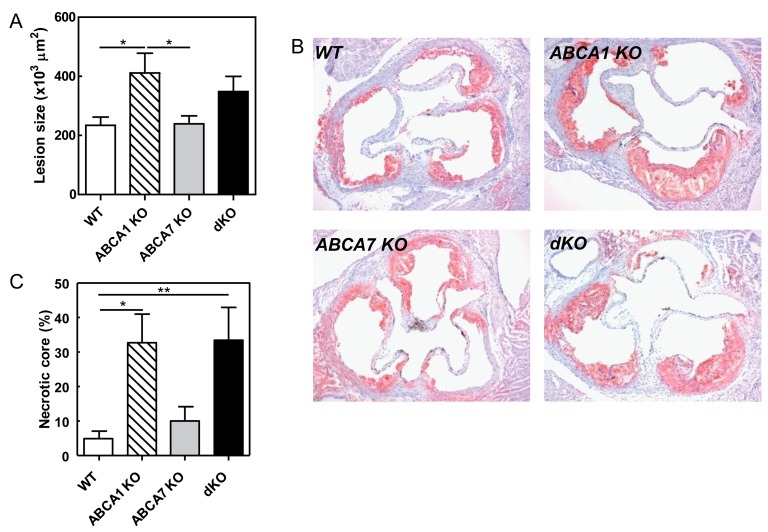


Figure 5 

**Figure:**
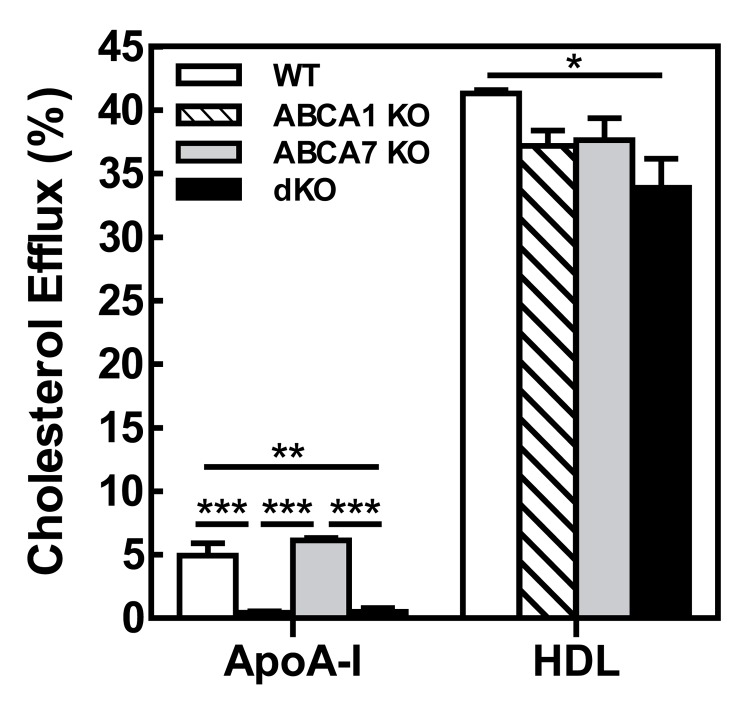


Figure 6 

**Figure:**
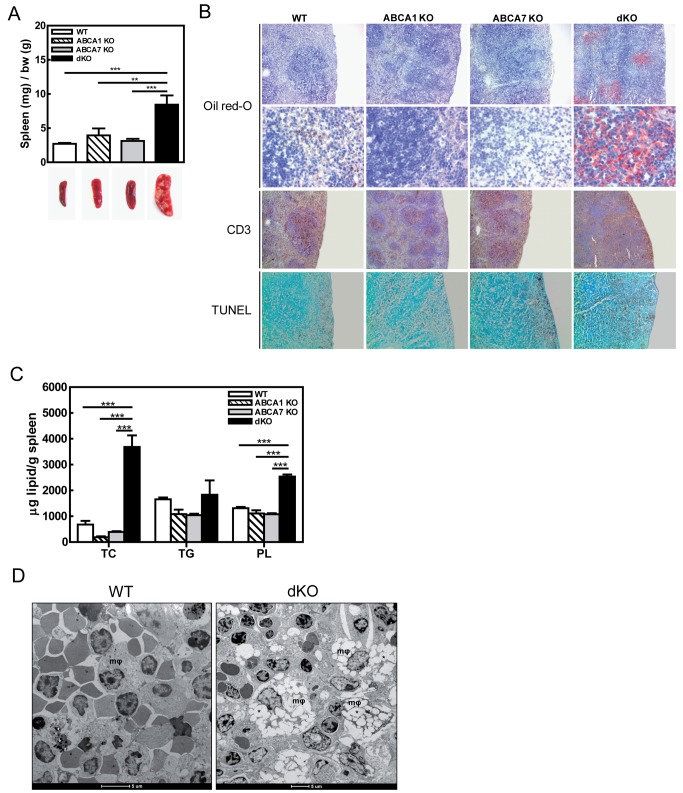


Figure 7 

**Figure:**
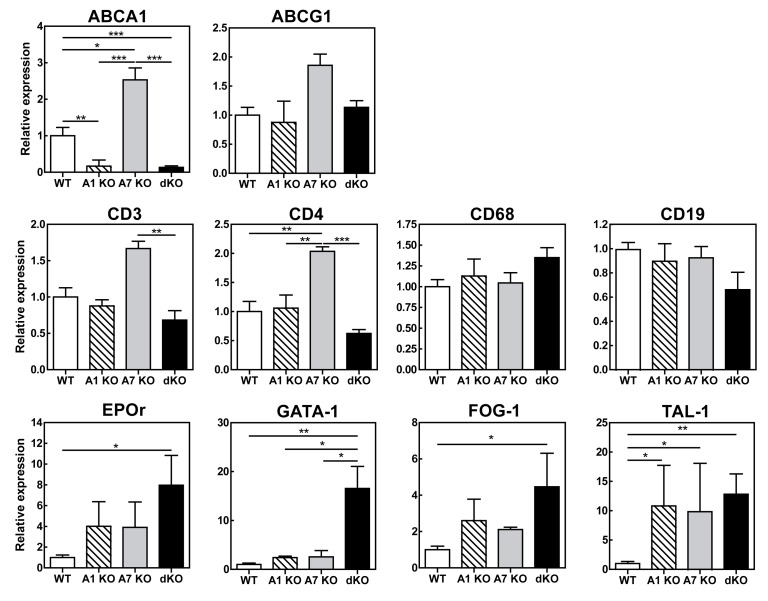


Figure 8 

**Figure:**